# Supplementary material for: A UMLS-based spell checker for natural language processing in vaccine safety
Source: BMC Med Inform Decis Mak. 2007 Feb 12;7:3. doi: 10.1186/1472-6947-7-3 (PMC1805499; doi:10.1186/1472-6947-7-3)
Supplement: Additional file 13 — RAP application directory. Sets up directory for RAP (RDF application for PHP) source code [file 1472-6947-7-3-S13.gz › rap/doc/tests/test_ManipulateMemModel.php.txt]

Test Manipulate and Search RDF Model

php
// Include RAP
define("RDFAPI\_INCLUDE\_DIR", "./../api/");
include(RDFAPI\_INCLUDE\_DIR . "RDFAPI.php");
// Filename of an RDF document
$base="example1.rdf";
// Create a new MemModel
$model = ModelFactory::getDefaultModel();
// Load and parse document
$model-load($base);
// Output model as HTML table
$model->writeAsHtmlTable();
echo "

";
// Ceate new statements and add them to the model
$statement1 = new Statement(new Resource("http://www.w3.org/Home/Lassila"),
new Resource("http://description.org/schema/Description"),
new Literal("Lassila's personal Homepage", "en"));
$statement2 = new Statement(new Resource("http://www.w3.org/Home/Lassila"),
new Resource("http://description.org/schema/Description"),
new Literal("Lassilas pers�nliche Homepage ", "de"));
$model->add($statement1);
$model->add($statement2);
$model->writeAsHtmlTable();
echo "

";
// Search model 1
$homepage = new Resource("http://www.w3.org/Home/Lassila");
$res = $model->find($homepage, NULL, NULL);
$res->writeAsHtmlTable();
echo "

";
// Search model 2
$description = new Resource("http://description.org/schema/Description");
$statement = $model->findFirstMatchingStatement($homepage, $description, NULL);
// Check if something was found and output result
if ($statement) {
echo $statement->toString();
} else {
echo "Sorry, I didn't find anything.";
}
echo "

";
// Search model 3
$res3 = $model->findVocabulary("http://example.org/stuff/1.0/");
$res3->writeAsHtmlTable();
echo "

";
// Write model as RDF
$model->writeAsHtml();
// Save model to file
$model->saveAs("Output.rdf");
?>
